# 

**Published:** 1900-03-03

**Authors:** 


					THE HOSPITAL. " The standard of highest purity.'1? Lancet. Marcii 3rd, 1900.
CADBURY'S PC* H CADBURY'S
cocoa m ^ ccocoa
ABSOLUTELY PURE.   W NO DRUGS OR ALKALIES USED.
No. 701>JVol. XXVII. IJfSSta Saturday,
March 3, 1900.
^SubscriptionTerms,] pRICE TWOPENCE.

				

